# “Death is certain, the time is not”: mortality and survival in *Game of Thrones*

**DOI:** 10.1186/s40621-018-0174-7

**Published:** 2018-12-10

**Authors:** Reidar P. Lystad, Benjamin T. Brown

**Affiliations:** 1 Australian Institute of Health Innovation, Faculty of Medicine and Health Sciences, Macquarie University, 75 Talavera Rd, Sydney, NSW 2109 Australia; 2 Faculty of Science and Engineering, Macquarie University, Sydney, Australia

**Keywords:** Mortality, Survival analysis, Violence, Prevention

## Abstract

**Background:**

*Game of Thrones* is a popular television series known for its violent and graphic portrayal of the deaths of its characters. This study aimed to examine the mortality and survival of important characters in *Game of Thrones*.

**Methods:**

Important characters appearing in Seasons 1 to 7 of *Game of Thrones* were included, and data on sociodemographic factors, time to death, and circumstances of death were recorded. Kaplan-Meier survival analysis with Cox proportional hazard regression modelling were used to quantify survival times and probabilities and to identify independent predictors of mortality, respectively.

**Results:**

Of the 330 characters that were included, 186 (56.4%) had died by the end of the study period. All but 2 deaths were due to injury, burns, or poisoning, with the majority being caused by assault (63.0%) or operations of war (24.4%). The survival time ranged from 11 s to 57 h and 15 min, with the median survival time estimated to be 28 h and 48 min. The probability of surviving at least 1 h in the show was 0.86 (95% CI 0.82 to 0.89). The analyses revealed worse survival for characters who were male (*P* < 0.001), lowborn (*P* < 0.001), had not switched allegiance during the show (*P* < 0.001), and who featured more prominently in the show (*P* < 0.001). After adjusting for other factors, whether or not a character had switched allegiance during the show and how prominently a character featured in the show were revealed to be independent predictors of death.

**Conclusions:**

The mortality risk is high among characters in *Game of Thrones*. The probability of dying within the first hour after first appearing on screen was about 14%. By the end of the seventh season, more than half of the important characters had died, with violent deaths being the most common by far. The probability of survival was worse for characters who were male or lowborn, who had not switched allegiance during the show, and who featured more prominently. There is great potential for preventing violent deaths in the world of *Game of Thrones*.

## Background

*Game of Thrones* is a popular HBO television series based on George R.R. Martin’s *A Song of Ice and Fire* book series (Martin 2012). The series portrays a fictional society characterised by political upheaval, civil wars, and wide-spread violence. Ceaseless feuding and the lack of stable democratic government precludes the establishment and development of institutions that can provide services to improve the health and well-being of the inhabitants. That the world of *Game of Thrones* is reminiscent of mediaeval European history is unsurprising considering that its creator has admitted that his creation was inspired by multiple historical events, in particular The Wars of the Roses which unfolded in England in the latter half of the fifteenth Century.

Violence has been a feature of every society throughout human history; however, there has been a sizable decline over time (Pinker 2011). For example, the annual rate of violent deaths of all kinds has declined from around 500 per 100,000 population in pre-state societies to 40 during the Middle Ages, and to less than 10 in modern societies (Pinker 2011; World Health Organization 2014). In pre-state societies, 15% of people died as a result of war, compared to less than 5 per 100,000 population today. In his book *The Better Angels of Our Nature: Why Violence Has Declined*, Steven Pinker identifies five historical forces that have contributed to the decline of violence in modern societies namely: the emergence of the modern nation-state with a monopoly on the legitimate use of force; the prioritisation of commerce over conquest; and a suite of enlightenment ideas (e.g. human rights, increased literacy, and the elevation of knowledge and rationality in problem-solving and decision-making) (Pinker 2011).

Few studies have investigated the health and well-being of the characters in *Game of Thrones*. One study described a dermatological disease called Greyscale (Lipoff 2016), while another study discussed forced fitness behaviours and associated hormonal responses (Rhodes and Zehr 2017). To the best of the authors’ knowledge, no epidemiological study of mortality and survival has been performed. The primary aim of this study was therefore to examine the mortality and survival of the characters in *Game of Thrones*. Specifically, to estimate survival time and probability, to identify predictive factors, and to describe causes and circumstances of deaths. The secondary aim was to give the authors an excuse to re-watch the first seven seasons before the final season reaches television screens worldwide.

## Methods

### Study population

This study included all important characters appearing in the first seven seasons of HBO’s *Game of Thrones* television series. An important character was defined as any individual who fulfilled each of the following criteria: human; listed in either the opening or closing credits; appeared on screen during current events (i.e. excluding flashbacks); and was not already deceased when first appearing on screen. Additional non-credited characters were included if they interacted with another character in a way that was either crucial to the storyline or character development. Having a speaking role was not an essential requirement because some characters were unable to speak for medical reasons (e.g. acquired brain injury and non-elective glossectomy).

### Data sources

The primary data source was the *Game of Thrones* DVD boxset, which included all 67 episodes from the seven seasons that have aired to date (Game of Thrones Seasons 1–7 [DVD] 2017). In addition, the Internet Movie Database (IMDb) and the Game of Thrones Wiki website were used to cross-check and validate information obtained through direct observation (Internet Movie Database (IMDb) [Internet] 2018; Game of Thrones Wiki [Internet] 2018). Both authors independently extracted sociodemographic, mortality, and time to event data. In the event of any discrepancies, both authors re-visited the original source material and resolved the discrepancy by mutual consensus.

The following sociodemographic variables were recorded for each character: sex, social status, type of occupation, religious affiliation, and allegiance. In regard to social status, characters who were lords, ladies, or legitimate offspring thereof were classified as “highborn”, whereas all other characters were classified as “lowborn”. Type of occupation was categorised as “silk collar” (e.g. clergy, merchants, politicians, and rulers) or “boiled leather collar” (e.g. warriors, farmers, and other occupations relying heavily on manual work). Because some characters switched allegiance during the show, both their last known allegiance and whether or not they switched allegiance during the show were recorded.

A proxy measure for how prominently a character featured in the show was created. This prominence score was calculated by taking the number of episodes that a character appeared in and dividing that by the number of total episodes that the character could have appeared in (i.e. the number of episodes occurring from the character first being introduced until the point of death or censoring). This ratio was then multiplied by the number of seasons that the character had featured in. The prominence scores were then categorised into tertiles (i.e. high, medium, and low).

### Outcome measures

The primary outcome for this study was time to death. The survival time was based on the duration, measured in hours, that a character survived after being introduced in the series. The net running time (i.e. running time excluding opening and closing credits) of all episodes in the seven seasons was summed to provide the cumulative net running time of the series, which denoted the maximum possible survival time prior to censoring. For each character, their start time was the time when they were first visualised/introduced in the series. The end time was either at the time of their death or at censoring if they remained alive until the end of the seventh season.

Although most deaths were visualised in unashamedly graphic detail on the screen, some deaths were evident only after being explicitly mentioned in the dialogue or implied by the storyline. For these characters, the time of death was assigned as the net running time at the end of the scene or episode in which their death was presumed to have occurred. For each death, the principal diagnosis, external cause of mortality, and place of occurrence were classified using the alphanumeric coding scheme of the International Statistical Classification of Diseases and Related Health Problems, 10th Revision, Australian Modification (ICD-10-AM) (National Centre for Classification in Health 2006), which is a derived version of the World Health Organization’s classification list. In addition, for each death the geographical location (i.e. Westeros, Essos, or Sothoryos), physical location (i.e. indoors or outdoors), and time of day (i.e. day or night) were recorded.

### Statistical analysis

Statistical analyses were conducted using R, version 3.3.2 (R Core Team. 2017). Summary statistics were used to describe the characteristics of characters and recorded deaths. Kaplan-Meier survival analysis was used to construct the curves that display the survival experiences of the cohort. Stratified analyses with the Peto-Peto test were used to compare survival experiences between subgroups of characters (i.e. by sociodemographic variables) (Peto and Peto 1972). Unlike the normal log-rank test which gives equal weight to all events of interest during the follow-up period, the Peto-Peto test assigns larger weights to hazards that occur early (Karadeniz and Ercan 2017). Furthermore, whereas the log-rank test assumes proportional hazards, the Peto-Peto test is more efficient when there is evidence of violation of the proportional hazards assumption (Karadeniz and Ercan 2017).

Univariable Cox proportional hazard regression models were used to identify potential predictor variables. Variables which showed *P* < 0.10 in the univariable model were included the subsequent multivariable Cox proportional hazard regression modelling. The proportional hazard assumption was evaluated by plotting Schoenfeld residuals as a function of time as described by Grambsch & Therneau (Grambsch and Therneau 1994). Where potential predictor variables displayed evidence of violation of the proportional hazards assumption, time dependent covariates were generated by creating interactions of the potential predictors and a function of survival time. These interaction terms were included alongside the time dependent covariates in the multivariable model. The final fitted multivariable model was used to identify independent predictors of survival and estimate hazard ratios (HR) with 95% confidence intervals (95% CI). Variables were considered independent predictors of survival when *P* < 0.05.

## Results

A total of 330 characters were included in this study, of which 237 (71.8%) were male, 226 (68.5%) were lowborn, and 196 (59.4%) were boiled leather-collar workers. See Table 1 for more details of the characteristics of the study cohort.

**Table 1:** Characteristics of important characters in *Game of Thrones* (*n* = 330)

Characteristic	Frequency (%)
Sex	
Male	237 (71.8)
Female	93 (28.2)
Social status	
Highborn	104 (31.5)
Lowborn	226 (68.5)
Type of occupation	
Silk collar	91 (27.6)
Boiled leather collar	206 (62.4)
Unknown/Unclear	33 (10.0)
Religion	
Great Stallion	19 (5.8)
Lord of Light	14 (4.2)
Faith of the Seven	35 (10.6)
Old Gods of the Forest	31 (9.4)
Drowned God	14 (4.2)
Other	13 (3.9)
Unknown/Unclear	204 (61.8)
Last known allegiance	
Stark	35 (10.6)
Targaryen	23 (7.0)
Night’s Watch	27 (8.2)
Lannister	25 (7.6)
Greyjoy	17 (5.2)
Bolton	13 (3.9)
Frey	17 (5.2)
Other	134 (40.6)
Unknown/Unclear	39 (11.8)
Allegiance switched during show	
No	285 (86.4)
Yes	45 (13.6)
Relative prominence of character	
Low	111 (33.6)
Medium	128 (38.8)
High	91 (27.6)
Season in which character first appeared	
1	101 (30.6)
2	52 (15.8)
3	47 (14.2)
4	36 (10.9)
5	40 (12.1)
6	48 (14.5)
7	6 (1.8)

By the end of the study period, 186 (56.4%) of the characters had died. The majority of deaths were injuries (*n* = 137; 73.7%), in particular wounds of the head and neck region, including 13 traumatic amputations at neck level (decapitations). The remainder of deaths were chiefly burns (*n* = 22; 11.8%) or poisonings (*n* = 9; 4.8%). Only two deaths from natural causes were recorded. The most common circumstances of deaths were assault (*n* = 116; 63.0%), operations of war (*n* = 45; 24.4%), and legal executions (*n* = 10; 5.4%). The majority of deaths occurred in Westeros (*n* = 147; 80.1%), while the most common place of occurrence was the home (*n* = 56; 30.4%). See Table 2 for a detailed overview of principal diagnoses, external causes of mortality, and places of occurrence.

**Table 2:** Characteristics of deaths of important characters in *Game of Thrones* (*n* = 186)

Characteristic	Frequency (%)
Principal diagnosis (ICD-10-AM code)^a^	
Injuries	
Open wound of head (S01)	2 (1.1)
Intracranial injury (S06)	10 (5.4)
Crushing injury of the head (S07)	1 (0.5)
Open wound of neck (S11)	26 (14.0)
Fracture of neck (S12)	3 (1.6)
Traumatic amputation at neck level (S18)	13 (7.0)
Open wound of chest (S21)	21 (11.3)
Open wound of abdomen, lower back and pelvis (S31)	6 (3.2)
Open wounds involving multiple body regions (T01)	25 (13.4)
Crushing injuries involving multiple body regions (T04)	2 (1.1)
Traumatic amputations involving multiple body regions (T05)	2 (1.1)
Injury of unspecified body region (T14)	26 (14.0)
Burns and corrosions	
Burns of multiple body regions (T29)	22 (11.8)
Poisoning by drugs, medicaments and biological substances	
Toxic effect of other and unspecified substances (T65)	9 (4.8)
Other and unspecified effects of external causes	
Asphyxiation (T71)	12 (6.5)
Maltreatment syndromes (T74)	4 (2.2)
Other ill-defined and unspecified causes of mortality (R99)	2 (1.1)
External cause (ICD-10-AM code)^a,b^	
Assault	
Assault by drugs, medicaments and biological substances (X85)	7 (3.8)
Assault by hanging, strangulation and suffocation (X91)	4 (2.2)
Assault by smoke, fire and flames (X97)	18 (9.8)
Assault by steam, hot vapours and hot objects (X98)	1 (0.5)
Assault by knife (X99.0)	54 (29.3)
Assault by other specified sharp object (X99.8)	11 (6.0)
Assault by blunt object (Y00)	2 (1.1)
Assault by pushing from high place (Y01)	4 (2.2)
Assault by bodily force (Y04)	5 (2.7)
Other maltreatment syndromes (Y07)	4 (2.2)
Assault by unspecified means (Y09)	6 (3.3)
Exposure to animate mechanical forces	
Bitten or struck by dog (W54)	3 (1.6)
Bitten or struck by other mammal (W55)	5 (2.7)
Intentional self-harm	
Intentional self-poisoning by and exposure to other unspecified drugs, medicaments and biological substances (X64)	2 (1.1)
Intentional self-harm by hanging (X70.0)	1 (0.5)
Intentional self-harm by knife (X78.0)	1 (0.5)
Intentional self-harm by jumping from a high place (X80)	1 (0.5)
Legal intervention and operations of war	
Legal execution (Y35.5)	10 (5.4)
War operations involving fires, conflagrations and hot substances (Y36.3)	3 (1.6)
War operations involving firearm discharge and other forms of conventional warfare (Y36.4)	42 (22.8)
Place of occurrence (ICD-10-AM code)^a,b^	
Home (Y92.0)	56 (30.4)
Prison (Y92.10)	2 (1.1)
Military camp (Y92.12)	15 (8.2)
Other specified institution and public administrative area (Y92.29)	16 (8.7)
Sports and athletics area (Y92.3)	1 (0.5)
Street and highway (Y92.4)	15 (8.2)
Trade and service area (Y92.5)	6 (3.3)
Large area of water (Y92.82)	6 (3.3)
Beach (Y92.83)	6 (3.3)
Forest (Y92.84)	11 (6.0)
Desert (Y92.85)	1 (0.5)
Other specified countryside (Y92.86)	22 (12.0)
Other specified place of occurrence (Y92.88)	11 (6.0)
Unspecified place of occurrence (Y92.9)	16 (8.7)
Geographical location	
Westeros	147 (80.1)
Essos	37 (19.9)
Location	
Indoors	74 (39.8)
Outdoors	89 (47.8)
Unknown/Unclear	23 (12.4)
Time of day	
Day	98 (52.7)
Night	57 (30.6)
Unknown/Unclear	31 (16.7)

^a^ICD-10-AM: International Statistical Classification of Diseases and Related Health Problems, 10th Revision, Australian Modification

^b^ICD-10-AM codes for external cause or place of occurrence were not assigned to deaths from natural causes (*n* = 2)

**Fig. 1:**
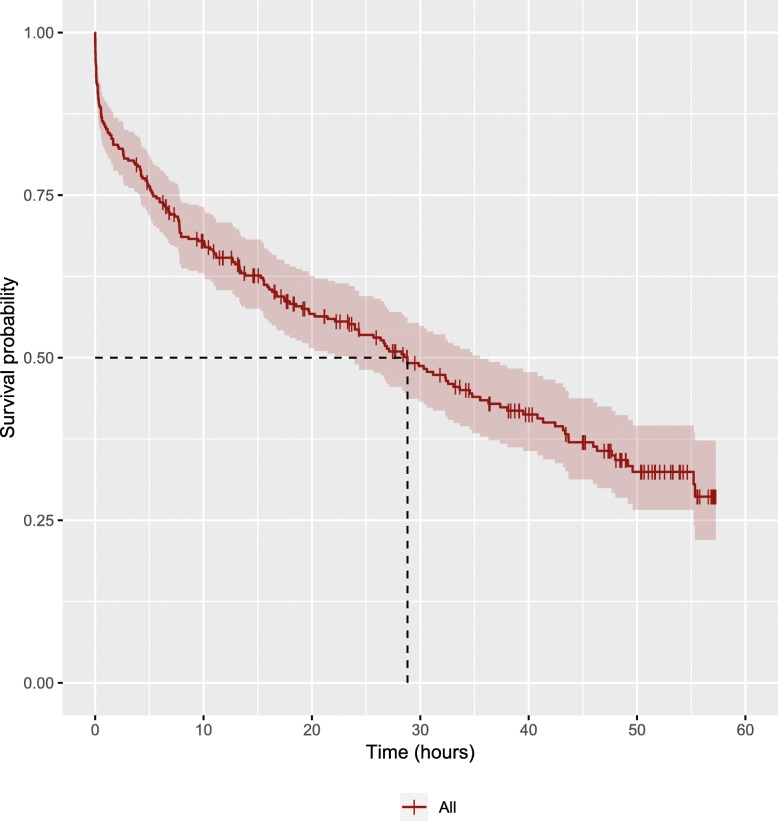
Survival curve for important characters in *Game of Thrones*

**Fig. 2:**
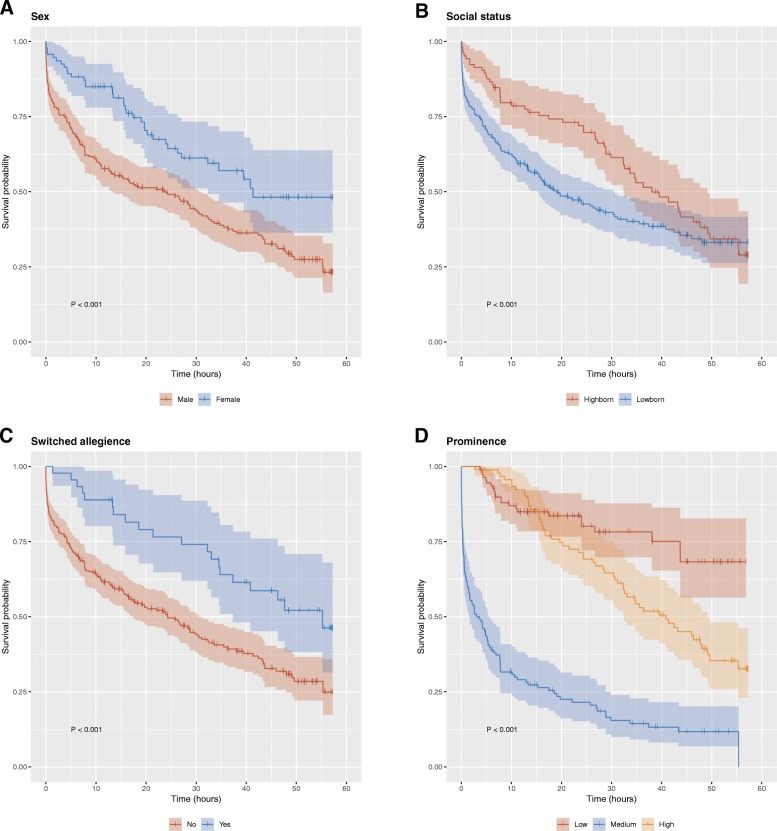
Survival curves for important characters in *Game of Thrones*, stratified by sex (**a**), social status (**b**), whether the character switched allegiance during the show (**c**), and how prominently the character featured in the show (**d**)

Figure 1 shows the survival curve for all important characters in the first seven seasons of the *Game of Thrones* television series. The survival time ranged from 11 s to 57 h and 15 min, with the median survival time estimated to be 28 h and 48 min. The probability of surviving at least 1 h in the show was 0.86 (95% CI 0.82 to 0.89). Figure 2 shows the survival curves stratified by sex (A), social status (B), whether the character switched allegiance during the show (C), and how prominently the character featured in the show (D). The stratified analyses revealed significantly shorter median survival times for males compared to females (24.0 h versus 41.3 h, respectively; *P* < 0.001), for lowborns compared to highborns (19.1 h versus 38.0 h, respectively; *P* < 0.001), and for those that did not switch allegiance compared to those that did (24.3 h versus 55.2 h, respectively; *P* < 0.001). In addition, there were significant differences in median survival time between characters whose prominence was low, medium, or high (*P* < 0.001).

**Fig. 3:**
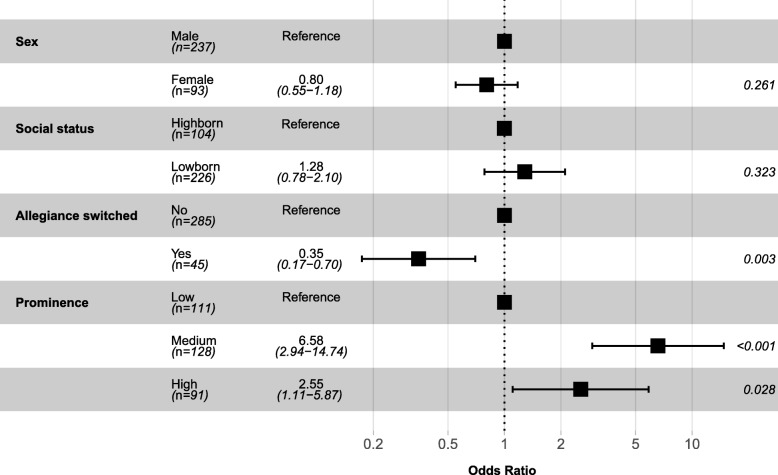
Forest plot of hazard ratios from multivariable Cox proportional hazard regression model

Figure 3 shows the findings from the final fitted multivariable Cox proportional hazard regression model. After accounting for other factors, how prominently a character featured in the show and whether or not they switched allegiance during the show were revealed to be independent predictors of death. Compared to characters who did not feature prominently in the show, the risk of death was more than 2.5 times greater for characters who featured very prominently (HR 2.55, 95% CI 1.11 to 5.87, *P* = 0.028) and more than 6.5 times greater than characters who only featured moderately prominently (HR 6.58, 95% CI 2.94 to 14.74, *P* < 0.001). Characters who switched allegiance during the show had significantly lower risk of death compared to those who did not switch allegiance (HR 0.35, 95% CI 0.17 to 0.70, *P* = 0.003).

## Discussion

This is the first scientific study to examine mortality and survival in *Game of Thrones*. It revealed that more than half of the important characters had died by the end of the seventh season, and the probability of dying within the first hour after first appearing on screen was about 14%. The vast majority of these deaths were due to injury, burns, or poisoning at the hands of other characters. The survival time of individual characters varied widely, a finding that echoes the quote from Jaqen H’ghar, an assassin featuring in *Game of Thrones*, which appears in the title of this paper.

The high rates of violent deaths observed in *Game of Thrones* are not without precedent in human history. For instance, archaeological evidence from prehistoric societies suggests that violent deaths accounted for about 15% of all-cause mortality, while ethnographic evidence indicates that the share of violent deaths for people living in pre-state societies was about 25% (Roser 2013a). In Europe, the homicide rates per 100,000 population have declined from around 40 homicides during the Middle Ages, to around 10 homicides in the eighteenth century, to around 3 homicides today (Roser 2013b; World Health Organization 2018).

Although many factors have undoubtedly contributed to the decline of violent deaths throughout human history, the following paragraph will be limited to a brief outline of some of the most significant ones. Perhaps foremost among these factors is the emergence of the nation-state with its monopoly on the legitimate use of force (Pinker 2011). The presence of such nation-states will tend to quell and restrain the anarchy, raiding, and feuding characterised by non-state societies. Next follows what Steven Pinker refers to as the “civilising process”, the process by which feudal territories were consolidated into large kingdoms with centralised authority and infrastructure for expanding commerce (Pinker 2011). As the importance of commerce increased, other people became more valuable alive than dead. The continued exchange of goods and services is, after all, more effective when trade partners are alive. The increased concern for the welfare of human beings, in turn, gives rise to the establishment of public institutions that can provide services to improve the health and well-being of the people (e.g. hospitals and public health departments). Lastly, but by no means least, is the rise of reason, the increasing application of knowledge and rationality in problem-solving and decision-making.

Where does the known world in *Game of Thrones* sit in regard to the factors that were highlighted in the preceding paragraph? For obvious reasons the world of *Game of Thrones* cannot be interpolated into human history; however, it may nevertheless be instructive to comment on to what extent any of the above-mentioned factors are apparent in *Game of Thrones*. Nation-states are not entirely universal in *Game of Thrones*, but they are common and wide-spread. The civilising process is clearly in progress, albeit far from completed. Although feudal territories in Westeros have coalesced into seven kingdoms, which in turn has been subjected to the rule of the Iron Throne, the political structure of the realm is evidently unstable. The legitimacy of the ruling power is questionable and the rule of law is ineffective, thus several competing factions are attempting to effect a regime change. There is clearly an emphasis on war rather than commerce, and although very limited medical care is available to some characters, there is a paucity of institutions for delivering public goods (e.g. schools, hospitals). Knowledge and reason do not appear to play a significant role in problem-solving and decision-making. Although some characters express concerns for human welfare and attempts to abolish slavery, such ideas are not universal or well enshrined in the world of *Game of Thrones*.

Given the societal structure outlined above, it should come as no surprise that violence prevention is presently not a priority in *Game of Thrones*. Despite being ubiquitous in human history, violence can be predicted and prevented (World Health Organization 2018). Effective violence prevention requires good quality data measuring the burden over time and identifying risk factors amenable to intervention. This study found that the risk of violent death was higher among characters who were male and lowborn. This is consistent with data from the real world suggesting that homicide rates are higher in countries and areas with lower socioeconomic status and that 80% of homicide victims are male (World Health Organization 2018). Other well-established risk factors for higher rates of violent deaths are transitions in political regime (World Health Organization 2018), the absence of good governance and the rule of law (World Health Organization 2018), and climatic instability (LeBlanc 1999; Jones et al. 1999). Although data pertaining to these risk factors were not recorded, it is nevertheless evident that the storyline in *Game of Thrones* is premised on the presence of these factors (e.g. the struggle to sit on the Iron Throne and that “Winter Is Coming”).

It could be argued there is great potential for violence prevention in the world of *Game of Thrones*. Beyond instituting legitimate (i.e. democratic) and stable governments that can deliver public goods such as justice based on the rule of law, the following recommendations could be offered: (1) increased efforts to expand commerce, thereby increasing the value of other people and creating more wealth and resources; (2) increased investment in institutions that can deliver public goods that will improve human health and well-being (e.g. schools, hospitals, and public health departments); (3) improve the built environment; and (4) develop and implement evidence-based violence prevention policies. Unlike violence prevention efforts in modern society, any of the above-mentioned recommendations could be implemented in *Game of Thrones* with the stroke of a pen. However, because this may negatively impact the show’s popularity, it seems unlikely any such changes will occur before the last episode of the final season reaches television screens worldwide.

### Limitations

Firstly, only important characters were included in this study, thus the findings may not be generalisable to the entire population in the world of *Game of Thrones*. Secondly, this study is potentially limited by the quality of data extraction and missing data. Independent data collection by two authors watching the DVD box set should have served to minimise errors. Furthermore, extracted data were subsequently cross-referenced with the information contained on the IMDb and the Game of Thrones Wiki site (Internet Movie Database (IMDb) [Internet] 2018; Game of Thrones Wiki [Internet] 2018). Although the accuracy of the information contained on the wiki site cannot be guaranteed, it is likely to be robust given that the information has been collaboratively reviewed by thousands of die-hard fans, including fans who have developed strong parasocial relationships, to the extent that overt grief was observed after the death of certain characters in the show (Daniel and Westerman 2017). Information regarding allegiance, occupation, and religion were indeterminable for some characters. Similarly, the unavailability of death certificates may have diminished the accuracy of the information regarding some deaths. Thirdly, it was not possible to calculate mortality rates per unit population because there were no available census data providing population estimates for the world of *Game of Thrones*. Lastly, the findings for time dependent covariates should be interpreted with caution. Although interaction terms were included alongside time dependent covariates in the multivariable Cox regression model, their parameter estimates are probably best interpreted as the average effect of the covariate (Allison 1995).

## Conclusions

This is the first scientific study to examine the mortality and survival in *Game of Thrones*. It revealed that the probability of a character dying within the first hour after first being introduced on screen was about 14%. By the end of the seventh season, more than half of the important characters had died, with violent deaths being the most common by far. The probability of survival was worse for characters who were male or lowborn, who had not switched allegiance during the show, and who featured more prominently in the show. There is great potential for preventing violent deaths in the world of *Game of Thrones*. Stable democratic governments, resilient institutions that deliver public goods, and implementation of evidence-based violence prevention polices can decrease the risk of violent deaths considerably.

## References

[CR1] Allison PD. Survival analysis using the SAS system: a practical guide. Cary, SAS Institute Inc; 1995.

[CR2] Daniel ES, Westerman DK (2017). Valar Morghulis (all parasocial men must die): having nonfictional responses to a fictional character. Commun Res Rep.

[CR3] Game of Thrones Seasons 1–7 [DVD]. United States: HBO; 2017.

[CR4] Game of Thrones Wiki [Internet]. San Francisco: Fandom; 2018. Available from: http://gameofthrones.wikia.com/wiki/Game_of_Thrones_Wiki

[CR5] Grambsch P, Therneau T (1994). Proportional hazards tests and diagnostics based on weighted residuals. Biometrika.

[CR6] Internet Movie Database (IMDb) [Internet]. Seattle: Amazon.com Inc.; 2018. Available from: http://www.imdb.com/

[CR7] Jones TL, Brown GM, Raab LM, McVickar JL, Spaulding WG, Kennett DJ (1999). Environmental imperatives reconsidered: demographic crises in western North America during the medieval climatic anomaly. Curr Anthropol.

[CR8] Karadeniz PG, Ercan I (2017). Examining tests for comparing survival curves with right censored data. Stat Transit.

[CR9] LeBlanc SA (1999). Prehistoric warfare in the American Southwest.

[CR10] Lipoff JB (2016). Greyscale–a mystery dermatologic disease on HBO's game of thrones. JAMA Dermatol.

[CR11] Martin GRRA (2012). Song of ice and fire series.

[CR12] National Centre for Classification in Health (2006). The international statistical classification of diseases and related health problems, tenth revision, Australian modification (ICD-10-AM) – tabular list of diseases.

[CR13] Peto R, Peto J (1972). Asymptotically efficient rank invariant test procedures (with discussion). J R Stat Soc Ser A Stat Soc.

[CR14] Pinker S. The better angels of our nature: why violence has declined. New York: Viking Books; 2011.

[CR15] R Core Team (2017). R: a language and environment for statistical computing, Version 3.3.2.

[CR16] Rhodes RE, Zehr EP. Fight, flight or finished: forced fitness behaviours in game of thrones. Br J Sports Med [Epub ahead of print 13 Sep 2017]. 10.1136/bjsports-2017-098170 PMC6579504 28903947

[CR17] Roser M. Ethnographic and archaeological evidence on violent deaths [internet]. Oxford: Out World in Data; 2013a. Available from: https://ourworldindata.org/ethnographic-and-archaeological-evidence-on-violent-deaths

[CR18] Roser M. Homicides [internet]. Oxford: Out World in Data; 2013b. Available from: https://ourworldindata.org/homicides

[CR19] World Health Organization (2014). Global status report on violence prevention.

[CR20] World Health Organization (2018). Violence info [Internet].

